# Long-term characterization of activated microglia/macrophages facilitating the development of experimental brain metastasis through intravital microscopic imaging

**DOI:** 10.1186/s12974-018-1389-9

**Published:** 2019-01-07

**Authors:** Sha Qiao, Yuan Qian, Guoqiang Xu, Qingming Luo, Zhihong Zhang

**Affiliations:** 1 Britton Chance Center for Biomedical Photonics, Wuhan National Laboratory for Optoelectronics-Huazhong University of Science and Technology, Room G304, Wuhan, 430074 Hubei China; 2 MoE Key Laboratory for Biomedical Photonics, School of Engineering Sciences, Huazhong University of Science and Technology, Wuhan, 430074 Hubei China

**Keywords:** Microglia/macrophage, Melanoma brain metastasis, Activation, Intravital imaging, MMP3

## Abstract

**Background:**

Microglia/macrophages (M/Ms) with multiple functions derived from distinct activation states are key surveillants maintaining brain homeostasis. However, their activation status and role during the brain metastasis of malignant tumors have been poorly characterized.

**Methods:**

Heterozygous CX3CR1-GFP transgenic mice were used to visualize the dynamic changes of M/Ms during the development of experimental brain metastasis through long-term intravital imaging equipped with redesigned bilateral cranial windows. The occurrence of experimental brain metastasis was evaluated after M/Ms were depleted with PLX3397, a CSF-1R inhibitor. The possible mediators of M/Ms in facilitating the brain metastasis were determined using reverse transcription-PCR, immunofluorescence, correlational analysis, and MMP inhibition.

**Results:**

Here, we showed that M/Ms were persistently activated and facilitated the formation of melanoma brain metastasis in vivo. We observed that M/Ms gradually and massively accumulated in the metastasis, with a 2.89-fold increase. To precisely depict the dynamic changes in the activation state of M/Ms, we defined the branching parameter to quantify their morphological alterations. The quantitative data showed that the extent of activation of M/Ms in metastatic foci was enhanced, with a 2.27-fold increase from day 1 to day 21. Along with the activation, the M/Ms increased their moving velocity (4.15-fold) and established a rapid, confined, and discontinuous motility behavior. The occurrence of melanoma brain metastasis was significantly hindered under M/M elimination, indicating the key role of M/Ms in the experimental brain metastasis. Interestingly, we found that M/Ms highly expressed matrix metalloproteinase 3 (MMP3), which were strongly correlated with M/M activation and the decrease of tight junction protein zonula occludens-1 (ZO-1). An MMP inhibitor moderately decreased the occurrence of melanoma brain metastasis, suggesting that MMP3 secreted by M/Ms may facilitate melanoma cell growth.

**Conclusions:**

Our results indicated that the activated M/Ms were essential in the development of melanoma brain metastasis, suggesting that M/Ms are a potential therapeutic target for tumor brain metastasis.

**Electronic supplementary material:**

The online version of this article (10.1186/s12974-018-1389-9) contains supplementary material, which is available to authorized users.

## Introduction

Brain metastasis often correlates with poor prognosis and high mortality [1–3]. Microglia are the main immune cells surveilling the brain environment to prevent pathogens and repair injuries [4–8]. However, studies focusing on the role of microglia/macrophages (M/Ms) in cerebral malignancy have mainly been restricted to primary brain tumors, such as glioma [9, 10]. The reaction of M/Ms in the brain metastasis of tumors from other organs and tissues (secondary brain tumors), which are much more common in the clinic [11, 12], remains poorly elucidated in vivo.

As brain-resident macrophages, M/Ms are highly plastic and function discriminatively in different physiological and pathological conditions [13, 14]. However, the reported function of M/Ms in brain metastasis has remained controversial because most studies have been performed in vitro. For instance, the microglia promoted the colonization of a breast cancer cell line through Wnt signaling in slice culture models [15]. In other studies, in vitro cultured microglia showed cytotoxicity to metastatic cancer cells [16, 17]. Thus, to directly reveal their function, we must focus on studying M/Ms in brain metastasis in vivo.

The functions of M/Ms are highly correlated with their activation state [13, 18]. Previous studies demonstrated that, after activation, the resting ramified M/Ms not only transformed their morphology to an amoeboid appearance [7, 19, 20], but also upregulated specific markers, such as translocator protein (TSPO), F4/80, and MHCII [21–23]. Accordingly, classical neuropathological studies have used immunostaining or morphological analysis to reveal the activation of M/Ms ex vivo. However, it is difficult to detect the dynamic changes in the morphology and motility behavior of M/Ms using these strategies; these dynamic changes are a direct reflection of both the activation state and the brain environment [24, 25]. Thus, monitoring dynamic changes in the morphology and motility behavior of M/Ms with long-term intravital microscopic imaging is a promising strategy for revealing their activation state and understanding their function during the development of brain metastasis.

Proteases such as matrix metalloproteinases (MMPs) often facilitate tumor cell migration by degrading dense stroma [26]. In previous studies of brain metastasis of breast and lung cancer, tumor cells were identified as the main destroyers of the blood brain barrier (BBB) via the production of MMPs (MMP1, MMP2, MMP3, and MMP9) [27–30]. However, the role of M/Ms in this process has seldom been studied. Moreover, in neurodegenerative diseases such as Parkinson’s disease, MMP3 released by damaged dopaminergic neurons activates M/Ms [31, 32]. Therefore, we hypothesized that M/Ms might be activated by MMP3, which is responsible for the disruption of the BBB, to facilitate the invasion of the brain parenchyma by metastatic cells.

In our study, we investigated the role of M/Ms in the brain metastasis of melanoma which is the most common malignant tumors diagnosed with brain metastasis [33, 34]. To directly observe M/Ms during brain metastasis, we used heterozygous CX3CR1-GFP transgenic mice in which all M/Ms constitutively expressed enhanced green fluorescent protein (EGFP) [24, 35, 36] and a red fluorescent protein (RFP)-expressing melanoma cell line (RFP-B16). We prepared an experimental brain metastasis model by stereotaxic injection [21, 37–39] and combined it with a redesigned bilateral cranial window in the skulls of CX3CR1-GFP transgenic mice (Additional file 1: Figure S1A) [40]. Through the bilateral windows in an individual mouse, the morphology and motility of M/Ms in the metastasis foci and normal brain regions were simultaneously monitored using confocal microscopy and conveniently compared throughout the whole term of melanoma brain metastasis. In addition, through an experimental brain metastasis model established by internal carotid injection (Additional file 1 : Figure S4A) [41], we analyzed the influence of M/Ms on melanoma brain metastasis and their potential regulatory mechanism. Taken together, these results demonstrated that M/Ms were persistently activated and facilitated the development of experiment brain metastasis partially because of their MMP3 expression.

## Materials and methods

### Mice and cell lines

Heterozygous CX3CR1-GFP transgenic mice (stock no. 005582) were purchased from The Jackson Laboratory (Bar Harbor, Maine, USA) and reproduced in a specific pathogen-free (SPF) animal facility in WNLO-HUST. C57BL/6 mice were purchased from Hunan Slack King of Laboratory Animal Co., Ltd. (Changsha, China). Female mice (8–12 weeks old) were used in all experiments. All animal studies were conducted in compliance with protocols approved by the Hubei Provincial Animal Care and Use Committee and in compliance with the experimental guidelines of the Animal Experimentation Ethics Committee of Huazhong University of Science and Technology. B16 melanoma cells were purchased from Wuhan Boster Biology Technology, Ltd. (Wuhan, China). Fluorescent B16 cells were obtained by transfection with a plasmid encoding RFP [42]. All cells were cultured in RPMI 1640 supplemented with 10% FBS (Thermo Scientific, Waltham, Massachusetts, USA) and 100 U/mL penicillin-streptomycin (Life Technologies, Carlsbad, California, USA) and were maintained in a 37 °C incubator (Thermo Scientific, Waltham, MA, USA) with 5% CO_2_.

### Establishment of melanoma brain metastasis

For the intracerebral model of brain metastasis [40], cranially opened animals were stereotactically injected with either 0.3 μL of PBS containing 1 × 10^3^ RFP-B16 cells or 0.3 μL of PBS alone using pulled glass micro-capillaries (VitalSense Scientific Instrument Co., Ltd., Wuhan, China) under the control of a PMI-100 pressure micro-injector (Dagan Ltd., Minneapolis, USA). Finally, the cranial chamber was sealed with a coverslip to perform intravital imaging procedures.

For the internal carotid model, mice were anesthetized by isoflurane and placed under a dissecting microscope as described previously [41]. In brief, the common carotid artery and internal carotid artery were prepared. Either 1 × 10^6^ B16 cells in 60 μL of PBS or PBS alone was injected through the carotid artery lumen to the internal carotid artery with a 31-G insulin syringe (catalog no. 324909, BD, USA). Melanoma brain metastasis was usually established at day 15 to 21.

### Animal preparation and intravital microscopic imaging

To observe the behavior of M/Ms in both the injection ipsilateral and contralateral sides of the brain in the same mice, we designed a bilateral cranial window. In brief, we shaved the heads of CX3CR1-GFP mice and removed a 1-cm^2^ flap of skin as described previously [43]. We marked a 2.5-mm circle on each side of the cortex, applied a thin layer of vetbond (3 M, MN, USA) on the area outside of the circle and used a dental drill (Ruiwode, Shenzhen, China) to thin the circumference of the circle. Then, the skull was gently opened and covered with a 3-mm coverslip. The optical window was sealed with 3 M vetbond followed by application of dental acrylic over the intact hemisphere.

On days 1, 5, 7, 14, and 21 after RFP-B16 inoculation, intravital imaging was performed using a PE spinning-disc confocal microscope (Perkin Elmer, USA) with dry 10× /0.3 NA and  20× /0.75 NA objectives (Olympus, Japan). The mice were anesthetized with 0.5–1.5% isoflurane in oxygen flow at 0.6 L/min controlled by a Matrix VMS small animal anesthesia machine (Midmark, Kettering, OH, USA). When beginning the imaging procedures, 60-μm-depth image stacks with 1.2-μm axial spacing were first acquired with a  10 × objective and then 20- to 30-min imaging sequences with an interval of 5 s were monitored using a  20 × objective. GFP and RFP fluorescent signals were separately excited by a DPSS laser (488 nm or 561 nm).

### Imaging data analysis

Image stacks and time series were analyzed using ImageJ (Version 1.49, Fiji) and Imaris (Version 7.6, Bitplane). The volume density of M/Ms was evaluated from fluorescent image stacks as previously described by counting the cells by the analysis volume (0.003125 mm^3^) [24]. The soma diameters of M/Ms were measured with Imaris, and the mean length of processes was calculated by ImageJ. The branching parameter was calculated by the mean soma diameter divided by the mean processes length. The trajectory of M/M somas was tracked via automatic spot analysis in Imaris, whereas the trajectory of M/Ms processes was tracked manually. The distributions of the trajectory, velocity, confinement ratio, and arrest coefficient were acquired in MATLAB (MathWorks, Natick, MA, USA). The confinement ratio was defined as the ratio of the displacement of a cell to the total length it traveled. The arrest coefficient was defined as the fraction of time that the velocity of cell was less than 2 μm/min.

### Selective depletion of M/Ms in vivo

M/Ms were selectively depleted as previously described [44]. In brief, mice were fed the indicated diets for 3 weeks before the melanoma cells were injected through the internal carotid vessel. The mice fed with the AIN-76A standard diet formulated with pexidartinib (PLX3397) (S7818, Selleck Chemicals, USA) at a dose of 290 mg/kg were denoted the PLX group, whereas mice given the AIN-76A standard diet without PLX3397 were denoted the normal group; the group of mice evaluated before being fed any diet was denoted the control group. While the brain metastases were being established, the mice were fed for another 15–21 days with the chow corresponding to their group. Then, the mice were sacrificed for further analysis.

### Immunofluorescence and histopathology

On days 1, 5, 7, 14, and 21 after melanoma cell inoculation, the mice were transcardially perfused with PBS, followed by 4% PFA in PBS. The brain tissues were dissected and dehydrated in 30% sucrose. Then, the tissues were embedded in Tissue-Tek OCT compound (Sakura Finetechnical Co., Ltd., USA) and sectioned into 10-μm slices on a Leica CM1950 cryostat (Wetzlar, Germany) at − 20 °C. The zonula occludens-1 (ZO-1) monoclonal antibody eFluor 570 (1:200, 41-9776-82, eBioscience, Thermo Fisher Scientific Inc. Waltham, MA, USA) was used to identify tight junctions in the brain. An anti-MMP3 antibody (1:1000, ab53015, Abcam, Cambridge, USA) and anti-rabbit Alexa fluor 647 (1:1000, #4414, Cell Signaling Technology, Danvers, MA, USA) were used to detect MMP3 expression on M/Ms. Anti-mouse F4/80 Alexa fluor 647 (1:100, 123122, BioLegend, San Diego, CA, USA) was used to evaluate the activation state of M/Ms in the brain tissues. All brain sections were imaged on a Zeiss LSM 710 confocal imaging system (Oberkochen, Germany). The data were analyzed with ImageJ (Version 1.49, Fiji).

For histopathology, fixed brain tissues were embedded in paraffin, sectioned, and stained with hematoxylin and eosin (HE). The HE sections were imaged on a Nikon Ni-E microscope (Nikon, Minato, Tokyo, Japan). All images were analyzed with ImageJ (Version 1.49, Fiji).

### Isolation of M/Ms

Deeply anesthetized mice were perfused with cold PBS as previously described [45]. Following perfusion, the mice were decapitated, and the brains were aseptically removed and stored in cold, serum-free medium with 10 U/mL papain (Roche, Switzerland) and 20 U/mL DNase I (Sigma-Aldrich, Merck, USA). Meninges from the brain were carefully removed with fine forceps, and the brain tissues were chopped and incubated in an incubator at 37 °C, 5% CO_2_ for 30 min with intermittent shaking every 5 min. Brain pieces were further triturated by repeated pipetting. Then, the suspensions were allowed to stand for 1 min, and the upper layer homogenate was passed through a 40-μm-nylon mesh to remove cell debris and undigested pieces. The lower layer was resuspended in a complete medium and passed through the nylon mesh again. The suspensions were centrifuged at 1000 g for 5 min at 4 °C. The resultant cell pellets were resuspended in 6 mL of 30% Percoll (GE, USA) and centrifuged at 500 g for 20 min at 4 °C. Then, the cell pellets were washed twice and seeded in 100-mm dishes for 30 min in an incubator. The adherent cells were collected to be identified with APC anti-mouse CD45 antibody (1:100, 103112, BioLegend, San Diego, CA, USA) and PE/Cy7 anti-mouse CD11b antibody (1:100, 101226, BioLegend, San Diego, CA, USA) with Guava easyCyte 8HT (Merck Millipore, Billerica, MA, USA) and used for RNA extraction.

### Reverse transcription-PCR

Total RNA of M/Ms was extracted with TRIzol® Reagent (Life Technologies, Carlsbad, CA, USA) according to the manufacturer’s protocol. Reverse transcription was performed with 500 ng of RNA in a 10-μL reaction solution of PrimeScriptTM RT Master Mix (Perfect Real Time) Sample (TaKaRa, Japan). PCR was performed with 28 cycles of denaturing (94 °C, 30 s), annealing (60 °C, 30 s), and extension (72 °C, 45 s), with a final extension at 72 °C for 7 min. PCR products were visualized by electrophoresis on a 1.5% (*w*/*v*) agarose gel containing 0.01% GelRed (Biosharp, Hefei, China) and imaged with a homemade fluorescence imaging system. Primer sequences were as follows: *β*-actin: forward primer 5′-GTGACGTTGACATCCGTAAAGA-3′ and reverse primer 5′-GCCGGACTCATCGTACTCC-3′; MMP3: forward primer 5′-TTGATGGGCCTGGAACAGTC-3′ and reverse primer 5′-AGTCCTGAGAGATTTGCGCC-3′; and MMP9: forward primer 5′-GGTCTTCCCCAAAGACCTGAAA-3′ and reverse primer 5′-GGGCACCATTTGAGTTTCCA-3′.

### MMP3 inhibition assay in vivo

To evaluate the role of MMP3 in melanoma brain metastasis development, we performed experiments as follows. The mice were inoculated with 1 × 10^6^ melanoma cells through the internal carotid vessel and treated daily with 0.5% carboxymethylcellulose (CMC) or 5 mg/kg of PD166793 ((S)-2-(4′-bromo-biphenyl-4-sulfonylamino)-3-methyl-butyric acid hydrate, SIGMA-ALDRICH, Merck, USA) dissolved in 0.5% CMC as described previously [27, 46]. The former group was denoted as the normal group, while the latter group was denoted as the PD group. After 21 days, the mice were perfused with PBS and 4% PFA. The mouse brains were collected and cryo-sectioned for MMP3 expression analysis.

### In vivo detection of the leakage of dextran after BM development

The mice were cranially opened and injected with B16-mCerulean cells. After the metastases developed on day 14, the mice were intravenously injected with dextran [47] (0.1 mL of 10 mg/ml 40 kD TMR-dextran, D1842, Thermo Scientific, Waltham, MA, USA) and were then monitored with PE spinning-disc confocal system to detect the signal of melanoma cells, M/Ms, and vessels. The images were processed with ImageJ (Version 1.49, Fiji).

### Statistical analyses

Linear charts, histograms, and correlation analysis were performed using GraphPad Prism (GraphPad Software, San Diego, CA, USA). For comparison between two groups, a two-tailed unpaired *t* test was used. For three or more groups, one-way ANOVA and the Kruskal-Wallis test were used between different time points. All data are presented as the mean ± SEM. For correlation analysis, Pearson’s correlation analysis was used. Differences between or among groups were indicated as follows: NS non-significant; *P < 0.05, **P < 0.01, ***P < 0.001, and ****P < 0.0001.

## Results

### M/Ms massively accumulated in the area of melanoma brain metastasis

To investigate the contribution of M/Ms in melanoma brain metastasis, M/Ms were monitored longitudinally in vivo. The CX3CR1-GFP mice were equipped with redesigned bilateral cranial windows and stereotactically injected with RFP-B16 (Additional file 1: Figure S1A). The window in which tumor cells or PBS was injected was denoted as the ipsilateral side, and the other window was denoted as the contralateral side (Additional file 1: Figure S1A). As the brain metastasis grew over the time extent, the tumor border observed with longitudinal imaging was not consistent, which indicated that it was not essential to discriminate the M/Ms in the tumor border or the core. Through this bilateral window model, the M/Ms in the microenvironment of brain metastasis and distant regions could be imaged simultaneously.

**Fig. 1:**
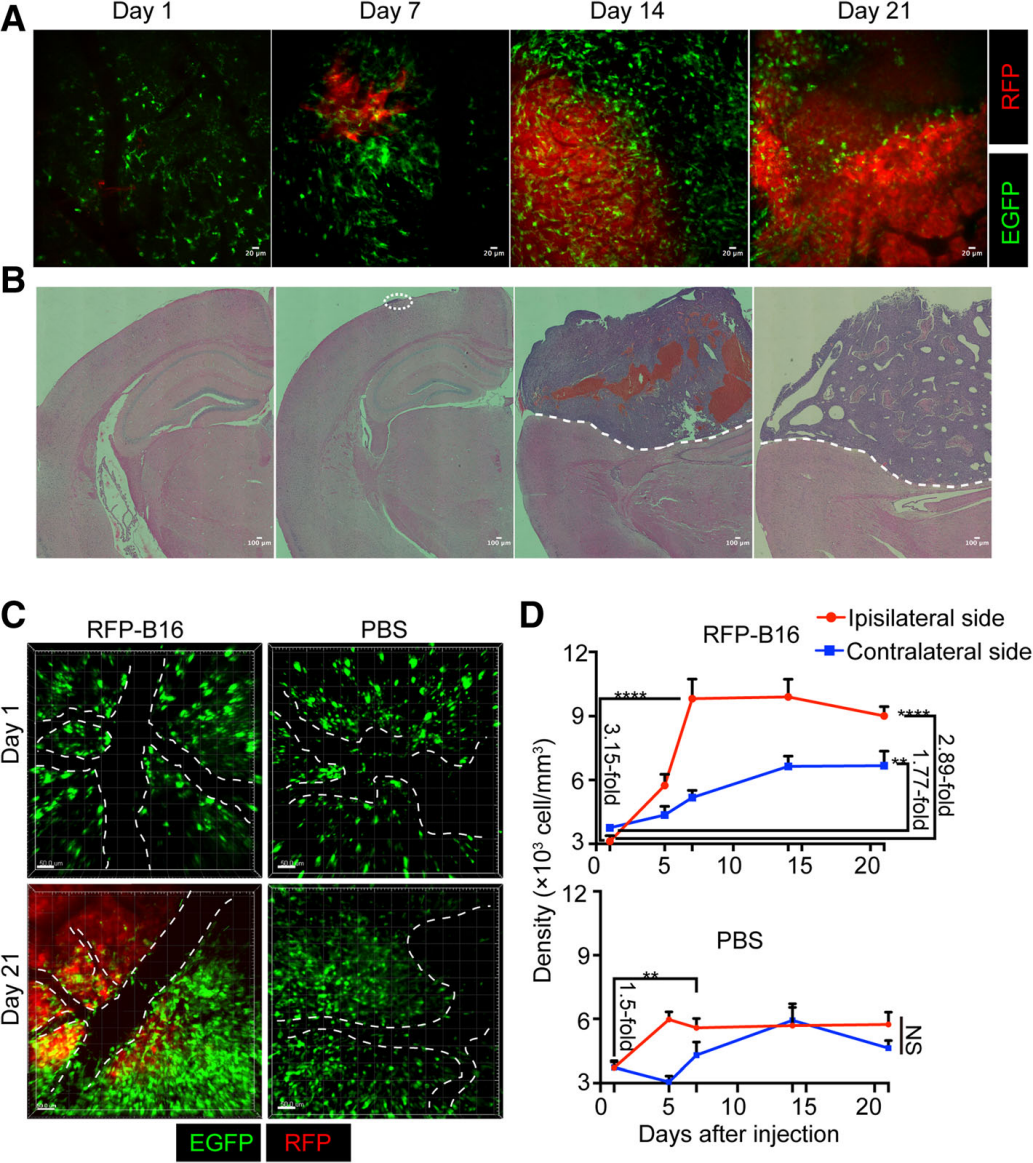
3D distribution of M/Ms during melanoma brain metastasis. **a** Intravital microscopic images of brains with melanoma metastasis in the same mice after RFP-B16 injection. Red: RFP, green: EGFP. Scale bar: 20 μm. **b** HE staining of brain tissue sections with metastasis after RFP-B16 injection. Scale bar: 100 μm. The white dotted line identifies the tumor area. **c** Representative results for the 3D distribution of M/Ms on day 1 and day 21 after RFP-B16 or PBS injection. The white dotted line refers to the lumen of blood vessels. Scale bar: 50 μm. **d** Volume density of M/Ms from the RFP-B16 (upper panel) or PBS (lower panel) injection group; *n* = 6 mice per group. The data are presented as the mean ± SEM

After tumor cell inoculation, we first monitored the growth of tumor cells in the brain. The results of intravital imaging showed that the tumor region expanded gradually (Fig. 1a), which was confirmed by HE staining of the brain tissue sections (Fig. 1b). Moreover, along with the enlargement of the metastasis, M/Ms massively infiltrated into the tumor area (Fig. 1a). To quantify the distribution of these cells, we acquired 3D images (60-μm depth) of both the ipsilateral and contralateral sides in the same mouse at days 1, 5, 7, 14, and 21 after melanoma cell or PBS injection (Fig. 1c and Additional file 1: Figure S1B). The fluorescent images showed that compared with the first day after injection, the population of M/Ms increased dramatically in the ipsilateral side of the RFP-B16 group at day 21 (Fig. 1c). Then, the cells were counted in five random cubes (250 μm × 250 μm × 50 μm) of every 3D image, and the cell number per cubic millimeter (volume density) was calculated. The data showed that in the ipsilateral side, the volume density of M/Ms increased to 3.15-fold that of day 1 on day 7 and then slightly decreased to 2.89-fold that of day 1 at day 21 after the inoculation of RFP-B16 (*P* < 0.0001, Fig. 1d, upper panel). Interestingly, in the contralateral side of the RFP-B16 group, the density increased slightly to 1.77-fold that of day 1 on day 21 (*P* < 0.01, Fig. 1d, upper panel), suggesting that the M/Ms in the distant region also sensed the metastasis. In the PBS control group, the density of M/Ms in the ipsilateral side increased to 1.5-fold that of day 1 on day 7 and remained at that level until day 21 (*P* < 0.01, Fig. 1d, lower panel). These results demonstrated that abundant M/Ms were accumulated in the metastatic foci and that injection of PBS also mobilized the M/M population; however, the effect of PBS was weaker than that induced by the injection of melanoma cells. In addition, in the contralateral side of the PBS group, the volume density of M/Ms did not differ significantly between time points (Fig. 1d, lower panel), indicating that these M/Ms were in a “resting” (inactive) state.

### Assessment of the dynamic change in activation of M/Ms in vivo with the branching parameter

**Fig. 2:**
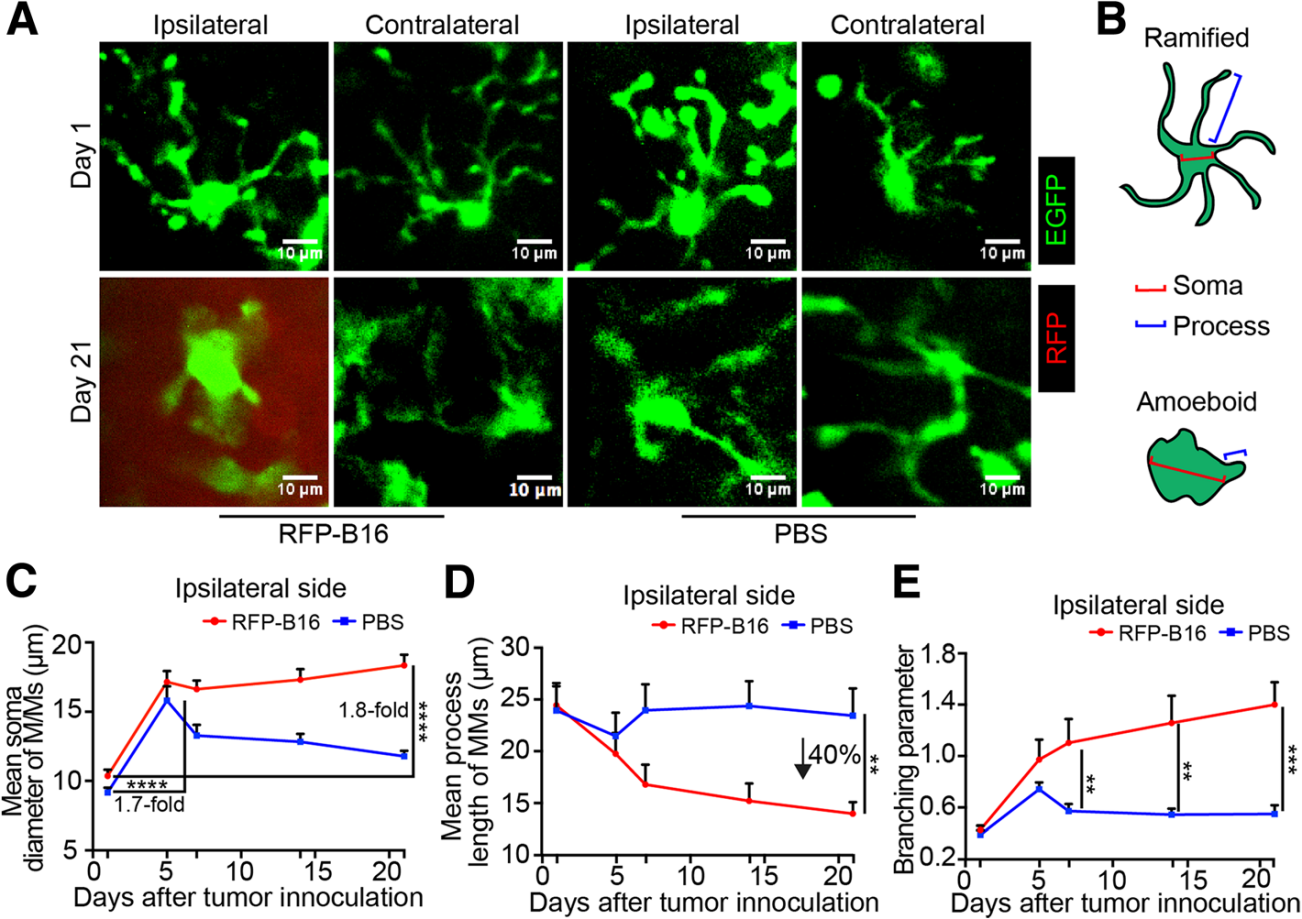
In vivo morphology analysis of M/Ms during the development of melanoma brain metastases. **a** Representative fluorescence images of the morphology of M/Ms in both sides on day 1 and day 21 after melanoma cell or PBS injection. Red: RFP, green: EGFP. Scale bar: 10 μm. **b** Schematic of the soma and processes of ramified or amoeboid M/Ms. **c** Mean soma diameter of M/Ms after RFP-B16 or PBS injection in the ipsilateral side. *n* = 15–35 cells at each time points from 6 mice per group. **d** Mean process length of M/Ms; *n* = 12–19 cells at each time point from 6 mice per group. **e** Branching parameter of M/Ms; *n* = 6 mice per group. The data are presented as the mean ± SEM

To further clarify the activation state of the accumulated M/Ms, their morphology was analyzed. As shown in Fig. 2a, at day 21, the M/Ms in the metastasis foci dramatically changed their appearance from ramified to an amoeboid shape compared with the cells at day 1, which indicated that the cells were absolutely activated. By contrast, the M/Ms in the contralateral side of the RFP-B16 group and ipsilateral side of the PBS group showed no obvious morphology changes on day 21 compared with day 1, which indicated they were not in an activated state (Fig. 2a).

Previous research often quantified M/M morphology based on soma diameter and process length [24, 48]. Thus, we measured these parameters of the M/Ms after a long-term intravital microscopic imaging on days 1, 5, 7, 14, and 21 (Fig. 2b). The results showed that 21 days after injecting RFP-B16 cells, the soma diameter of M/Ms in the ipsilateral side increased sharply to 1.8-fold that of M/Ms. on day 1 (*P* < 0.0001, Fig. 2c). By contrast, the process length of those M/Ms decreased to only 60% of that of the M/Ms in the PBS group (on day 21, *P* < 0.005, Fig. 2d). In the contralateral side, the M/Ms showed a slightly increased soma diameter but an unchanged process length (Additional file 1: Figure S2A-B). Interestingly, the soma of M/Ms was also obviously enlarged before day 5 in the ipsilateral side after PBS injection (1.7-fold, *P* < 0.0001) but showed a recurrence thereafter (Fig. 2c). These data indicated that the activated M/Ms displayed enlarged soma and shortened processes.

To more directly depict the activation status of the cells, we defined the branching parameter as the mean soma diameter divided by the mean process length. In general, M/Ms in which the process length is shorter than twice the soma diameter are classified as being in an activated state, whereas M/Ms in which the process length is more than twice the soma diameter are classified as resting cells [25, 49]. Thus, an increase in the branching parameter beyond 0.5 indicated enhancement of the extent of activation. By contrast, a decrease in the parameter approaching 0.5 indicated that the M/Ms tended to be in the resting status. As the branching parameter combines both information on soma diameter and processes length, it more precisely depicts the activation state of the M/Ms (Fig. 2c, d, and e). As shown in Fig. 2e, the branching parameter of the M/Ms was substantially higher (day 7: 1.10, 1.92-fold, day 14: 1.259, 2.30-fold, day 21: 1.4, 2.54-fold) in the ipsilateral side of the RFP-B16 group than that of the PBS group. In addition, the upregulation of F4/80 on M/Ms in the region of the metastasis instead of the non-tumor region also indicated the activated state of these cells (Additional file 1: Figure S2D). The obvious higher expression of Iba1 also clarified the highly activated state of M/Ms in BM mouse brains [50], and the colocalization of CD206 reflected the M2-like activation of these cells [51], which indicated their possible pro-tumoral function (Additional file 1: Figure S2E). Besides, the branching parameter of the M/Ms from the contralateral side of the RFP-B16 group and from the ipsilateral side of the PBS group increased to 0.7524 and 0.7415 on day 5 but then tended to decrease to 0.5 (the resting state) on day 21 (Fig. 2e and Additional file 1: Figure S2C). These data indicated that the M/Ms were sensitive to the surrounding microenvironment and the activated M/Ms induced by the different stimuli (PBS injection and RFP-B16 cells) could be distinguished according to the branching parameter. Hence, these results revealed that during melanoma brain metastasis, the extent of activation of M/Ms in the metastatic foci was strongest among the M/Ms from different regions or groups, and this activated status was maintained until day 21.

### Activated M/Ms in brain metastases established a rapid, confined, and discontinuous movement pattern

**Fig. 3:**
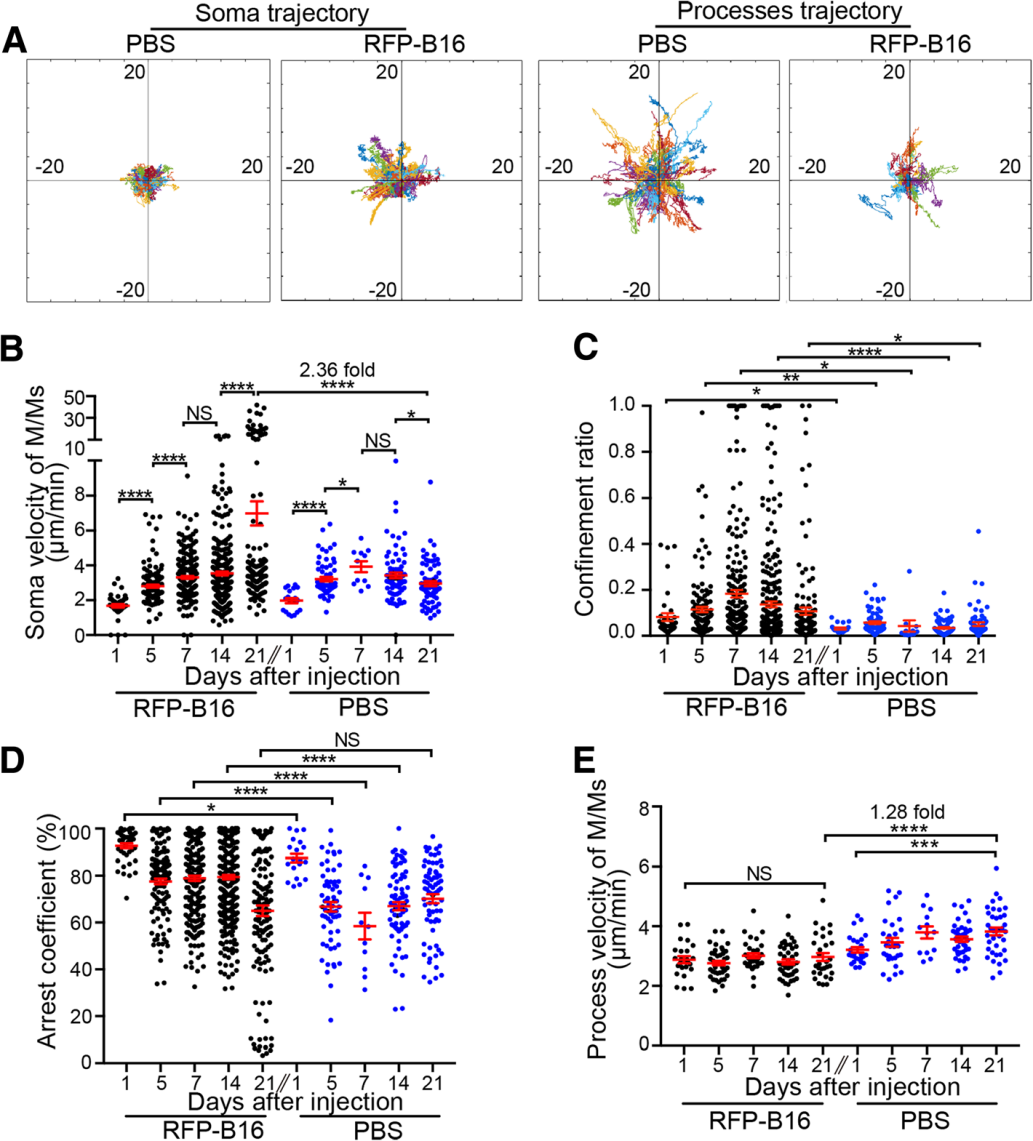
Distinct dynamics of M/Ms during the development of melanoma brain metastases. **a** Trajectory of soma or processes in M/Ms on day 21 from the PBS or RFP-B16 injection group. **b**–**d** The velocity (**b**), confinement ratio (**c**), and arrest coefficient (**d**) of M/M somas on days 1, 5, 7, 14, and 21 after RFP-B16 or PBS injection in the ipsilateral side. Each dot represents a single cell soma. **e** Velocity of M/M processes after RFP-B16 or PBS injection in the ipsilateral side. Each dot represents a single cell process. The data are presented as the mean ± SEM

Then, we further characterized the dynamic behavior of the activated M/Ms during the development of melanoma brain metastasis. As shown in the movies and track plots, at day 21 after melanoma cell inoculation, the trajectories of M/M soma movement were obviously broader than those in the PBS group (Fig. 3a and Additional file 2: Supplemental movie).

The motility of M/Ms in both groups was characterized in terms of related dynamic parameters, such as the mean velocity, confinement ratio, and arrest coefficient [42, 52, 53]. The mean velocity represents the moving speed of the soma or processes of M/Ms. The confinement ratio is the ratio of the displacement of an M/M soma to the total length it has traveled. The arrest coefficient is the fraction of time that an M/M soma is pausing. The soma velocity of M/Ms in the ipsilateral side showed a gradual increase from 2.81 μm/min at day 1 and reached 6.99 μm/min at day 21 (Fig. 3b). By contrast, the soma velocity of M/Ms in the same side of the PBS group first increased to 3.93 μm/min (from day 1 to day 7) but then recovered to 2.96 μm/min at day 21. Finally, the soma velocity of M/Ms in metastases was 2.36-fold higher than that in the ipsilateral side of the PBS group (day 21, *P* < 0.0001, Fig. 3b). In addition, the M/Ms in the metastatic foci exhibited a higher confinement ratio at each time point than those in the PBS group (Fig. 3c). Moreover, the M/Ms in the RFP-B16 group were more arrested than those in the PBS group at days 1, 5, 7, and 14, but decreased to the same level as the PBS group at day 21 (Fig. 3d). These results indicated that after sensing the changes in the brain stroma, the M/Ms moved restrictively in the metastatic foci and often paused to seek information from melanoma cells. Then, the M/Ms gradually increased the moving speed of the somas, possibly due to their activation. That is, the activated M/Ms established a rapid, confined, and discontinuous movement pattern in the metastatic foci.

Interestingly, the process velocity of M/Ms in the metastases remained slow and barely changed during melanoma brain metastasis (Fig. 3e). This result was consistent with their amoeboid morphology (shrinking process) and trajectories (Fig. 2a, b and Fig. 3a, Additional file 2: Supplemental movie). In addition, the M/Ms in the contralateral side of both the PBS and RFP-B16 groups showed no obvious differences in the soma and process mean velocity (Additional file 1: Figure S3A-B).

### M/Ms were necessary for the formation of melanoma brain metastasis

**Fig. 4:**
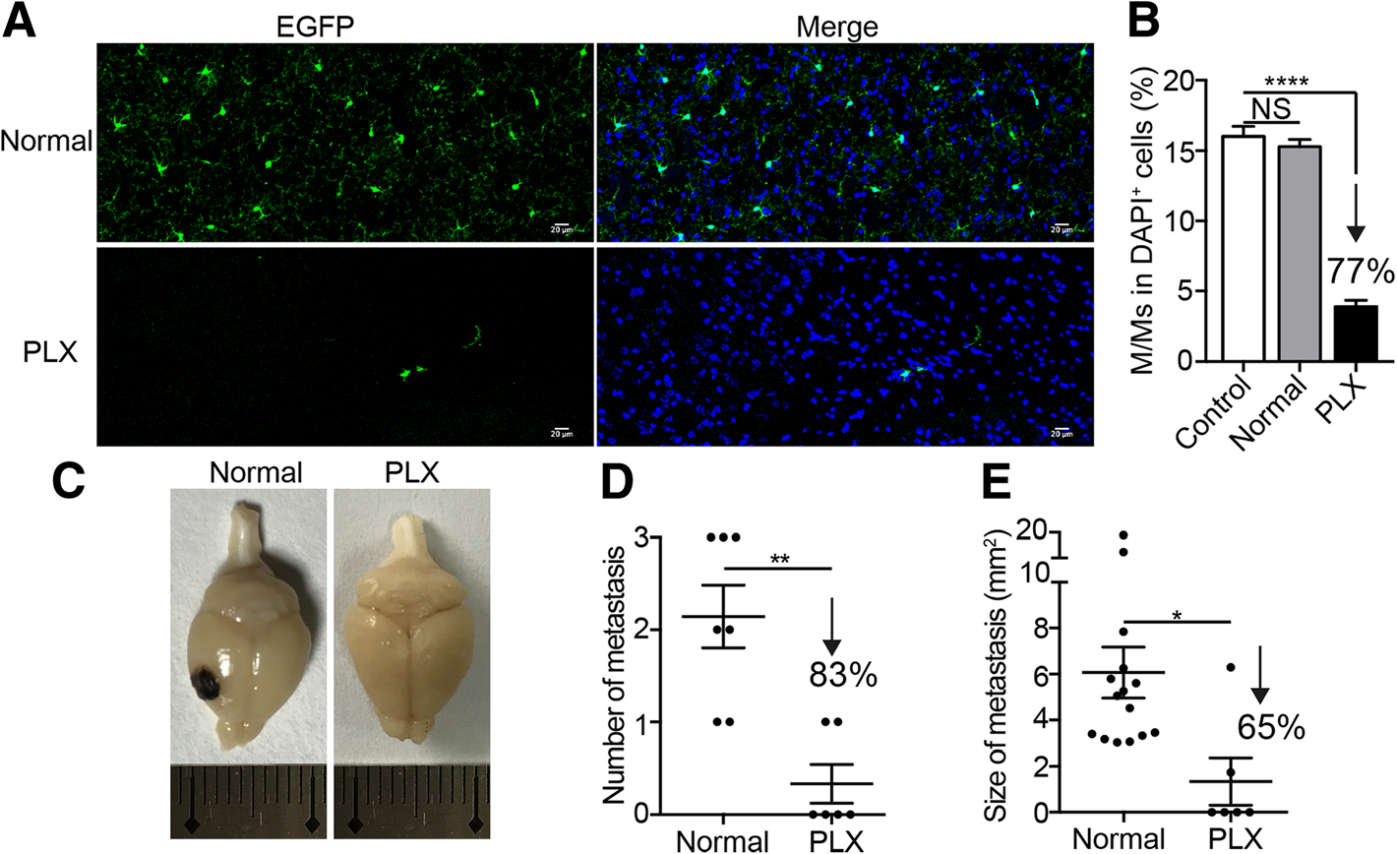
The role of M/Ms in the development of melanoma brain metastases. **a** Representative fluorescent images of the presence of M/Ms after specific depletion by PLX3397. Normal: mice fed with standard diet without PLX3397. PLX: mice fed with standard diet formulated with PLX3397. Green: EGFP, blue: DAPI. Scale bar: 20 μm. **b** Quantitative results for the fluorescent images shown in (**a**). Control: mice before feeding with any diet; *n* = 25 images from 3 mice per group. **c** Representative photograph of brain tissues of mice after M/M elimination. **d**–**e** The number (**d**) and size (**e**) of melanoma brain metastases in the indicated groups; *n* = 7 mice in the normal group, *n* = 6 mice in the PLX-treated group. The data are presented as the mean ± SEM

To further verify whether M/Ms play a key role in brain metastasis, we established another animal model of melanoma brain metastasis through internal carotid injection. First, we confirmed that there were no significant differences in the formation of metastasis between the transgenic (TR) mice and wild type (WT) C57BL/6 mice (Additional file 1: Figure S4A-C). Subsequently, a small-molecule inhibitor of colony stimulating factor-1 receptor (CSF-1R), PLX3397, was used as previously described to specifically delete the M/Ms before tumor cell inoculation [36, 44, 54]. Confocal fluorescent imaging results of brain cryo-sections showed that after 21 days of administration of PLX3397, 77% of M/Ms were eliminated (Fig. 4a, b and Additional file 1: Figure S4D). Then, to record the number and size of the metastases, the PLX3397-treated mice were injected with B16 melanoma cells through the internal carotid, and their brains were dissected 3 weeks after injection. Brain metastases were only detected in two of six M/M-depletion mice but in all of the normal mice (Fig. 4c, d). In addition, the total number and mean size of the brain metastases decreased 83 and 65%, respectively, after most of the M/Ms were depleted (Fig. 4d, e). These data revealed that M/Ms were necessary for the development of melanoma brain metastasis.

### MMP3 expressed by M/Ms contributed to M/M activation and affected the tight junctions of the BBB

Brain metastasis formation is accompanied by the disruption of the BBB [3, 55–59], which can be attributed to a variety of MMPs, such as MMP1, MMP2, MMP3, and MMP9 [60, 61]. In addition to degrading the BBB, MMP3 is also considered a strong M/M activator [31, 32]. Therefore, we tested whether the M/Ms expressed MMP3 to benefit their activation and affect the integrity of the BBB to facilitate the growth of metastatic cancer cells.

**Fig. 5:**
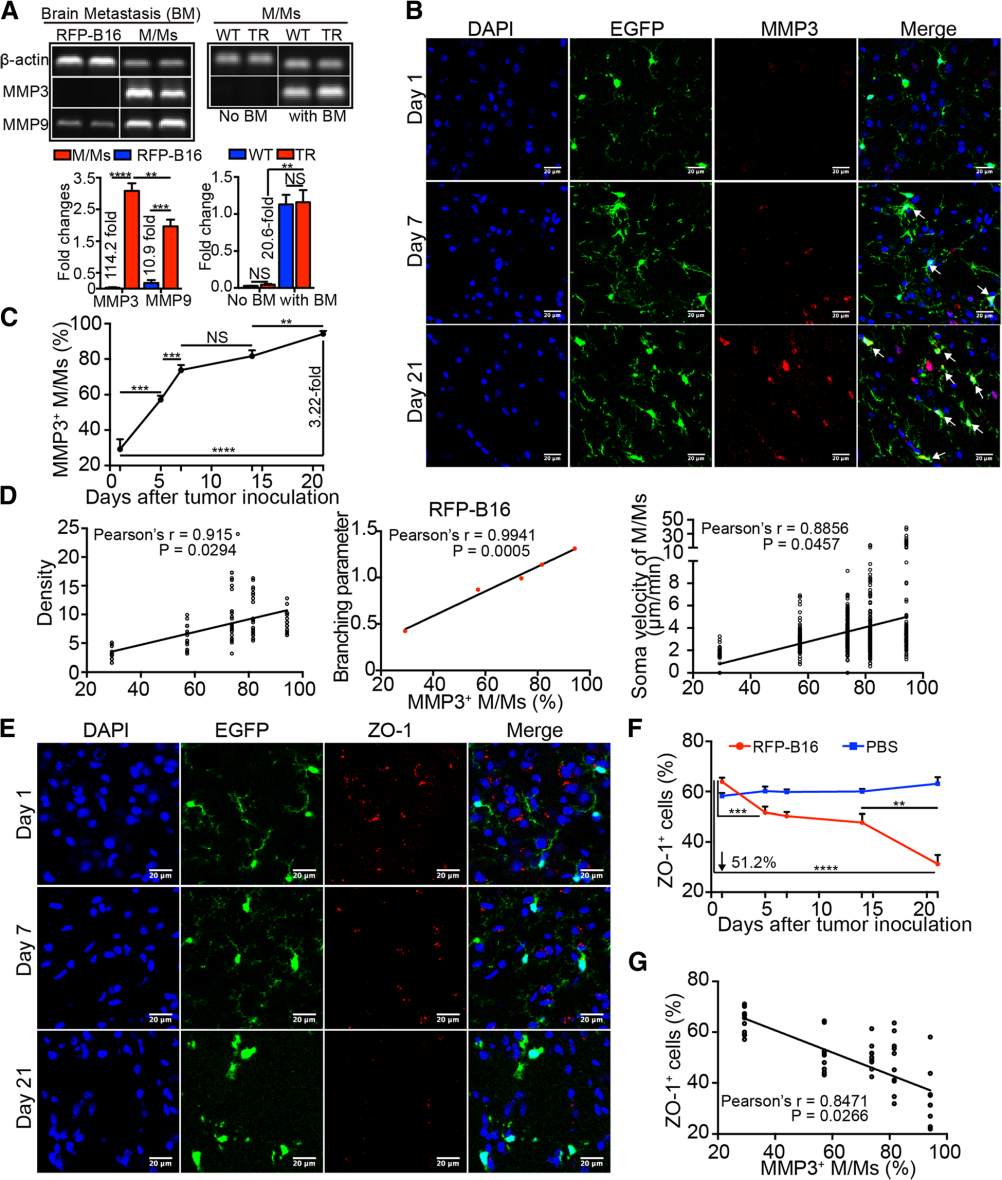
MMP3 expression in M/Ms contributed to their activation and disruption of the tight junction. **a** Agarose gel electrophoresis results of RT-PCR for detecting the expression of MMPs and the quantitative analysis. WT: C57BL/6 mice. TR: CX3CR1-GFP mice. *n* = 3 mice per group. **b** Representative immunofluorescence images of MMP3 in M/Ms. Blue: DAPI, green: EGFP, red: MMP3. Scale bar: 20 μm. **c** Quantitative analysis of the images shown in (**b**); *n* = 10 images from 3 mice per time point. **d** Correlation analysis between the volume density, branching parameter, soma velocity of M/Ms and the proportion of MMP3^+^ M/Ms in the RFP-B16 injection group; *n* = 6 mice per group. The data are presented as the mean ± SEM. **e** Representative immunofluorescence results for ZO-1 expression in the brain. Blue: DAPI, green: EGFP, red: ZO-1. Scale bar: 20 μm. **f** Quantitative results for the proportion of ZO-1^+^ cells shown in (**f**); *n* = 10 images from 3 mice per time point. **g** Correlation analysis between the proportion of MMP3^+^ M/Ms and the proportion of ZO-1^+^ cells. The data are presented as the mean ± SEM

To detect MMP expression, M/Ms were isolated from the brains with metastases. The flow cytometry results confirmed that 90% of the isolated cells were M/Ms (CD11b^high^CD45^low^, Additional file 1: Figure S5A). Surprisingly, reverse transcription-PCR (RT-PCR) results showed that M/Ms from metastatic brains expressed both MMP3 and MMP9, whereas RFP-B16 tumor cells barely expressed MMP3 but produced low levels of MMP9 (Fig. 5a, upper panel). Compared with the expression of MMPs by tumor cells, MMP3 expression by M/Ms was 114.2-fold higher (*P* < 0.0001), while MMP9 production was only 10.9-fold higher (*P* < 0.001, Fig. 5a, lower panel). The M/Ms showed a higher level of MMP3 than MMP9 (1.57-fold, Fig. 5a, lower panel). Moreover, compared with normal brains, MMP3 expressed by M/Ms was highly upregulated in brains with metastasis (20.6-fold, *P* < 0.01, Fig. 5a, right panel). In addition, no significant difference in the MMP3 expression of M/Ms was observed between WT and transgenic mice (Fig. 5a, right panel). Then, we detected MMP3 production by M/Ms during the development of metastasis through immunofluorescence. As shown in Fig. 5b, c, the proportion of MMP3^+^ cells in M/Ms (white arrow) increased from 29.9% (day 1) to 94.2% (day 21) after tumor cell inoculation (3.22-fold, *P* < 0.0001).

To investigate whether the activation state of M/Ms was affected by MMP3, the relationship between MMP3 expression and activation state was analyzed in M/Ms. The results showed that the distribution, morphology, and motility of M/Ms were all highly correlated with their MMP3 expression in the RFP-B16 injection group (ipsilateral side, Pearson’s correlation coefficient = 0.915, 0.9896, 0.8856, *P* = 0.0294, 0.0013, 0.0457 for volume density, branching parameter, and soma velocity, respectively Figure 5d). However, no significant correlation was observed in the M/Ms from the PBS injection group (Additional file 1: Figure S5B). These results suggested the possibility of the participation of MMP3 expressed by M/Ms in the activation of M/Ms during melanoma brain metastasis.

Then, to explore if the MMP3 expressed by M/Ms disrupted the tight junction of the BBB to promote brain metastasis of melanoma cells, the tight junction protein ZO-1 was detected via immunofluorescence staining. As shown in Fig. 5e, f, the proportion of ZO-1^+^ cells decreased by half (from 63.92% at day 1 to 31.21% at day 21, *P* < 0.0001, Fig. 5f) after melanoma cell inoculation. ZO-1 expression was not changed in the control PBS group (Fig. 5f and Additional file 1: Figure S5C). Moreover, the decrease in ZO-1 expression was correlated with MMP3 upregulation in M/Ms (Pearson’s correlation coefficient = 0.8471; *P* = 0.0266. Fig. 5g). Additionally, the leakage of dextran from the cortical vessels was also observed in BM-bearing mice in vivo (Additional file 1: Figure S5D). Hence, these results indicated that the tight junction of the BBB was gradually disrupted during the formation of melanoma brain metastasis, which is possibly attributed to the MMP3 in M/Ms.

### MMP3 inhibition hindered tight junction impairment and melanoma brain metastasis

**Fig. 6:**
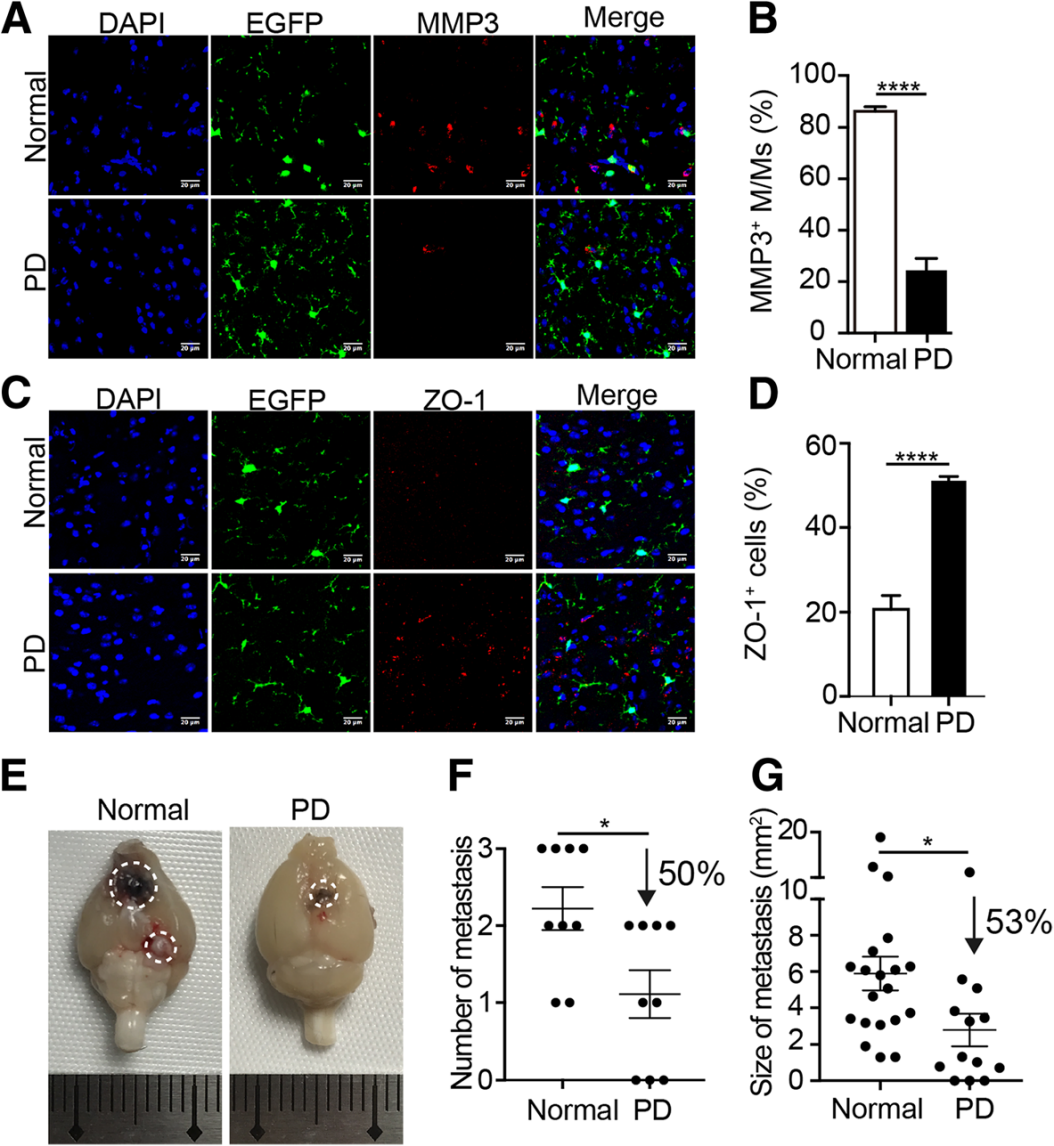
Verification of the role of MMP3 in melanoma brain metastases. **a** Representative immunofluorescence images of MMP3 expression by M/Ms after PD166793 treatment. Normal: mice treated with 0.5% CMC without PD166793. PD: mice treated with PD166793 dissolved in 0.5% CMC. Scale bar: 20 μm. **b** Quantitative results for the proportion of MMP3^+^ M/Ms shown in (**a**); *n* = 13 images from the normal group and 9 images from the PD group, 3 mice per group. **c** Representative immunofluorescence image of ZO-1 expression in the brain after MMP3 inhibition. Scale bar: 20 μm. **d** Quantitative results for the proportion of ZO-1^+^ cells shown in (**c**); *n* = 12 images from the normal group and 7 images from the PD group, 3 mice per group. **e** Representative photograph of brain tissues after PD166793 treatment. **f**–**g** The number (**f**) and size (**g**) of melanoma brain metastases in the indicated groups; *n* = 9 mice per group. The data are presented as the mean ± SEM

To further confirm the role of MMP3 in tight junction disruption and facilitation of melanoma brain metastasis, mice were treated daily with PD166793 [27], an MMP inhibitor that is known to decrease MMPs activity, after tumor cell inoculation. The immunofluorescence staining and quantitative data indicated that after inhibition, only 24% of M/Ms expressed MMP3, whereas the proportion of MMP3^+^ M/Ms in the normal mouse group was 86% (Fig. 6a, b). Interestingly, compared with that in normal brains, ZO-1 expression was substantially higher in the brains of mice treated with PD166793 (Fig. 6c). Quantitatively, the proportion of ZO-1^+^ cells in the PD166793-treatment group was 2.47-fold higher than that in normal mice (*P* < 0.0001, Fig. 6d). These data indicated that the impairment of the tight junction of the BBB was prevented by MMP3 inhibition. Subsequently, we evaluated the formation of melanoma brain metastases. As shown in Fig. 6e, f, and g, compared with the values in the normal mouse group, the total number and mean size of metastases in the PD166793-treatment group decreased by 50 and 53%, respectively. Taken together, these results demonstrated that the facilitation of melanoma brain metastasis formation by M/Ms was partially due to the crucial role of MMP3 in BBB impairment.

## Discussion

Tumor-associated macrophages (TAMs) often polarize to an M2 phenotype with tumor-promoting functions [62]. However, as brain-resident macrophages, the role of M/Ms in brain metastasis has been poorly characterized. Here, with long-term intravital microscopic imaging, we identified that the M/Ms were persistently activated with distinct cellular dynamics and facilitated the formation of melanoma brain metastasis, which were highly correlated with their MMP3 expression.

Long-term intravital imaging data indicated that the M/Ms immediately sensed and then distinguished the physical stimulus (PBS injection) and biological stimulus (RFP-B16 cells). Their different activation states could be precisely and intuitively evaluated by the branching parameter in vivo. After the injection of PBS or RFP-B16, the branching parameter of M/Ms in the ipsilateral side increased immediately to more than 0.5, indicating M/M activation (Fig. 2e). Moreover, the branching parameter of the M/Ms in the RFP-B16 group increased to 1.4 at day 21, whereas that of the M/Ms in the PBS group recovered to 0.5 (Fig. 2e). These data indicated that the M/Ms in the metastatic foci strengthened their extent of activation, whereas the M/Ms stimulated by PBS injection returned to the resting state, further supporting the strong applicability of the branching parameter in depicting cell activation. Moreover, the activation state of M/Ms characterized by other parameters, including population and motility behavior (soma velocity, confinement ration, and arrest coefficient), was consistent with that indicated by the branching parameter (Fig. 1 and Fig. 3). Together, these intravital longitudinal imaging results demonstrated the sensitivity and persistent activation of M/Ms during the development of melanoma brain metastasis. In addition, according to the Ki67 detected in Additional file 1: Figure S2E, portions of these cells may originate from themselves through proliferation; however, the possibility that portions of these cells originated from the periphery should be further investigated through bone marrow chimeras [63].

M/Ms show high plasticity in different microenvironments [4, 18, 64]. In our model, M/Ms were necessary in the occurrence of melanoma brain metastasis. Combined with the colocalization of Iba1 and M2-type marker CD206 in M/Ms from BM-bearing mice, these data suggested that they might polarize to a phenotype with the function of promoting melanoma brain metastasis. After 77% of the M/Ms were deleted by a CSF-1R inhibitor (PLX3397), the number and size of the metastases decreased 83 and 65%, respectively (Fig. 4c–e). By isolating the M/Ms from the brains with metastasis, we detected that M/Ms expressed high levels of MMP3. In addition, the expression of MMP3 in M/Ms was gradually upregulated along with the development of the metastasis. Furthermore, the MMP3 upregulation highly correlated with the M/M activation state and loss of a tight junction protein (ZO-1) in the brain (Fig. 5). Although the ZO-1 expression recovered after inhibition of MMP3 with PD166793, moderate inhibition of melanoma brain metastasis was observed (Fig. 6c–g). However, the reason why the MMP3 expression was decreased after treated with PD166793 is not clear, while the inhibition of MMP3 activity by PD166793 may offer one possible explanation for their effect on BM decrease [27]. These results revealed that the facilitation of melanoma metastasis by M/Ms was partially attributable to their MMP3 expression. Together, these data indicated the role of M/Ms in melanoma brain metastasis and suggested that MMP3 of M/Ms related pathway is a promising therapeutic target.

Furthermore, the mechanisms driving the activation of M/Ms and the upregulation of their MMP3 expression remain to be elucidated. The M/Ms displayed a rapid but confined, discontinuously motility pattern (Fig. 3), which indicated the possibility of information exchange during melanoma brain metastasis. There might be other elements that can induce changes in the movement of these cells and their activation. For example, there are often massive numbers of dead cells and debris in the tumor microenvironment. In our study, apoptotic neuron cells were surrounded by Iba1^+^ M/Ms in the BM tissues, which indicated that the death of neurons may correlate with the activation of M/Ms (Additional file 1: Figure S2F). Therefore, it is possible that the M/Ms were stimulated by the damage associated molecular pattern (DAMP) signals released by damaged neuronal cells or dead melanoma cells [65]. With regard to MMP3 inhibition, future efforts should focus on targeted delivery of the inhibitor to M/Ms through biocompatible carriers, such as lipid-peptide nanoparticles [66–68]. Moreover, mice with specific MMP3 deficiency in M/Ms could also be used to further clarify its effects on M/Ms activation in BM [32, 69]. These investigations would further verify the role of MMPs from M/Ms in brain metastasis and might enable greater therapeutic effects.

## Conclusions

In summary, our study demonstrated that the M/Ms showed massive accumulation during the development of melanoma brain metastasis. Through long-term intravital microscopic imaging, we also provided dynamic information for precisely depicting the activation status and motility behavior of the M/Ms to reveal their different responses to distinct PBS or melanoma cell stimuli. Moreover, we demonstrated that these activated M/Ms facilitated melanoma brain metastasis in vivo, which were highly correlated with their MMP3 expression. Taken together, these results indicate a crucial role of M/Ms in melanoma brain metastasis and suggest them as a promising therapeutic target in tumor brain metastasis.

## Additional files

Additional file 1: **Figure S1.** The melanoma brain metastasis established by stereotactically injection. **Figure S2.** The activation state of M/Ms in melanoma brain metastasis. **Figure S3.** Velocity of M/Ms after RFP-B16 or PBS injection in the contralateral side. **Figure S4.** The melanoma brain metastasis established by internal carotid injection. **Figure S5.** The effect of MMP3 in M/Ms on their activation and the disruption of BBB integrity. (DOCX 14768 kb)

Additional file 2: EGFP^+^ M/Ms motility on day 21 after PBS or RFP-B16 injection. The images were captures at 5 s/frame. The video is played at 100 times real speed. The colorful line refers to the trajectories of M/M soma and processes during the movie. Gree, EGFP^+^ M/Ms; red, RFP-B16 cells. Scale bar, 10 μm. (MPEG 35564 kb)

## Abbreviations

BBB: Blood brain barrier
CMC: Carboxymethylcellulose
CSF-1R: Colony stimulating factor-1 receptor
DAMP: Damage-associated molecular pattern
EGFP: Enhanced green fluorescent protein
HE: Hematoxylin and eosin
M/Ms: Microglia/macrophages
MHC: Major histocompatibility complex
MMP: Matrix metalloproteinase
PBS: Phosphatic buffer solution
RCR: Polymerase chain reaction
RFP: Red fluorescent protein
SPF: Specific pathogen-free
TAM: Tumor-associated macrophages
TR: Transgenic
TSPO: Translocator protein
WT: Wild type
ZO-1: Zonula occludens-1
