# Supplementary material for: Long-term characterization of activated microglia/macrophages facilitating the development of experimental brain metastasis through intravital microscopic imaging
Source: J Neuroinflammation. 2019 Jan 7;16:4. doi: 10.1186/s12974-018-1389-9 (PMC6323850; doi:10.1186/s12974-018-1389-9)

**Long-term characterization of activated microglia/macrophages facilitating the development of experimental brain metastasis through intravital microscopic imaging**

Sha Qiao^†,‡,1^, Yuan Qian^†,‡,1^, Guoqiang Xu^†,‡^, Qingming Luo^†,‡^ and Zhihong Zhang^†,‡,^*

^†^ Britton Chance Center for Biomedical Photonics, Wuhan National Laboratory for Optoelectronics-Huazhong University of Science and Technology, Wuhan, Hubei 430074, China

^‡^ MoE Key Laboratory for Biomedical Photonics, Huazhong University of Science and Technology, Wuhan, Hubei 430074, China

Email address:

Sha Qiao, shaq@hust.edu.cn

Yuan Qian, joker@hust.edu.cn

Guoqiang Xu, xuguoqiang@hust.edu.cn

Qingming Luo, qluo@mail.hust.edu.cn

^*^Correspondence: Zhihong Zhang, czyzzh@mail.hust.edu.cn

Address: Room G304, Britton Chance Center for Biomedical Photonics, Wuhan National Laboratory for Optoelectronics-Huazhong University of Science and Technology, Wuhan 430074, China. Fax: +86-27-87792034; Tel.: +86-27-87792033;

Additional file 1:

Supplemental Figure 1. The melanoma brain metastasis established by stereotactically injection. (A) The schema of intravital microscopic imaging the brain of CX3CR1-GFP mice. (B) Representative results of 3D distribution of M/Ms during the melanoma brain metastasis. Green: EGFP. Scale bar: 50 μm.

Supplemental Figure 2. The activation state of M/Ms in melanoma brain metastasis. (A) Mean soma diameter of M/Ms after RFP-B16 in the contralateral. Resting M/Ms represented the M/Ms in the contralateral side of PBS injection; n = 15-35 cells in each time points from 6 mice per group. (B) Mean process length of M/Ms; n = 12-19 cells at each time point from 6 mice per group. (C) Branching parameter of M/Ms; n = 6 mice per group. The data are presented as the mean±SEM. (D) Representative immunofluorescence results for the detection of F4/80 expressed on M/Ms. Blue: DAPI; green: EGFP; red: F4/80; grey: RFP-B16. Scale bar: 20 μm. (E) Immunohistochemical labeling of activated M/Ms with Iba1 and CD206, and the detection of proliferation with Ki67. The black asterisk indicates the region that labeled. BM: brain metastases. Scale bar: 50 μm. (F) Representative images of TUNEL in NeuN-positive cells and the surrounding Iba1 expression M/Ms. The arrow label displays the activated M/Ms. Blue: DAPI; red: NeuN; green: TUNEL. BM: brain metastases. Scale bar: 30 μm.

Supplemental Figure 3. Velocity of M/Ms after RFP-B16 or PBS injection in the contralateral side. (A-B) Velocity of M/Ms soma (A) or processes (B) on days 1, day 5, day 7, day 14 and day 21 after RFP-B16 or PBS injection in contralateral side. Every dot represented as one soma or process of M/Ms. n = 6 mice per group. Data are presented as the mean±SEM.

Supplemental Figure 4. The melanoma brain metastasis established by internal carotid injection. (A) The schema of internal carotid injection. (B-C) The number (B) and size (C) of melanoma brain metastasis from WT and TR mice separately. WT, C57BL/6 mice. TR, CX3CR1-GFP mice. n = 10 mice per group. (D) Representative fluorescent images for the detection of the presence of M/Ms in control group. Data are presented as the mean±SEM.

Supplemental Figure 5. The effect of MMP3 in M/Ms on their activation and the disruption of BBB integrity. (A) Identification of M/Ms isolated from mouse brains. (B) Correlation analysis between the volume density, branching parameter, soma velocity of M/Ms and the proportion of MMP3^+^ M/Ms in the PBS injection group; n = 6 mice per group. The data are presented as the mean±SEM. (C) Representative immunofluorescence results for the detection of ZO-1 expression in the brain on day 1, day 7, day 21 after PBS injection. Blue: DAPI; green: EGFP; red: ZO-1; Scale bar: 20 μm. (D) *In vivo* imaging of the leakage of dextran from the cortical vessels in control and BM-baring mice within 30 min of intravenously administrated TMR-dextran. Blue: B16-mCerulean; red: dextran; green: EGFP; scale bar: 50 μm.

Additional file 2:

Supplemental movie. EGFP^+^ M/Ms motility on day 21 after PBS or RFP-B16 injection. The images were captures at 5 sec/frame. The video is played at 100 times real speed. The colorful line refers to the trajectories of M/M soma and processes during the movie. Gree, EGFP^+^ M/Ms; red, RFP-B16 cells. Scale bar, 10 μm.

Supplemental Figure 1


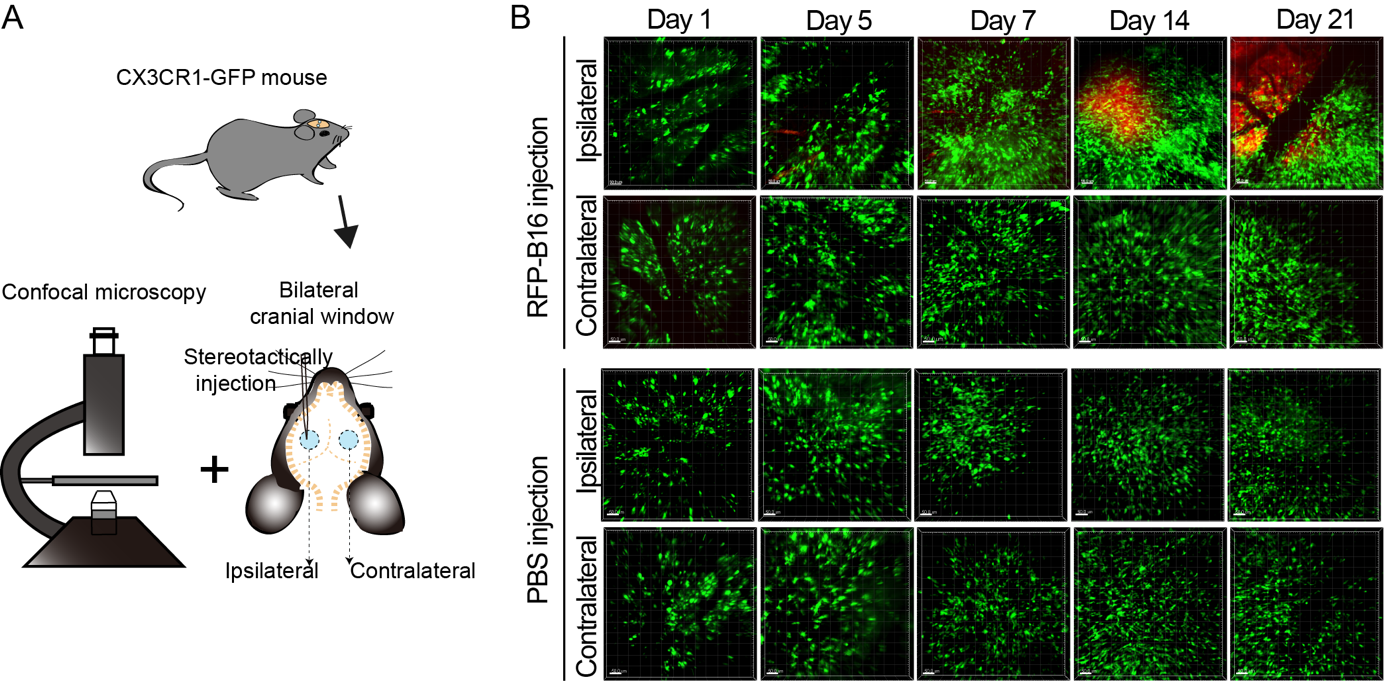


Supplemental Figure 2


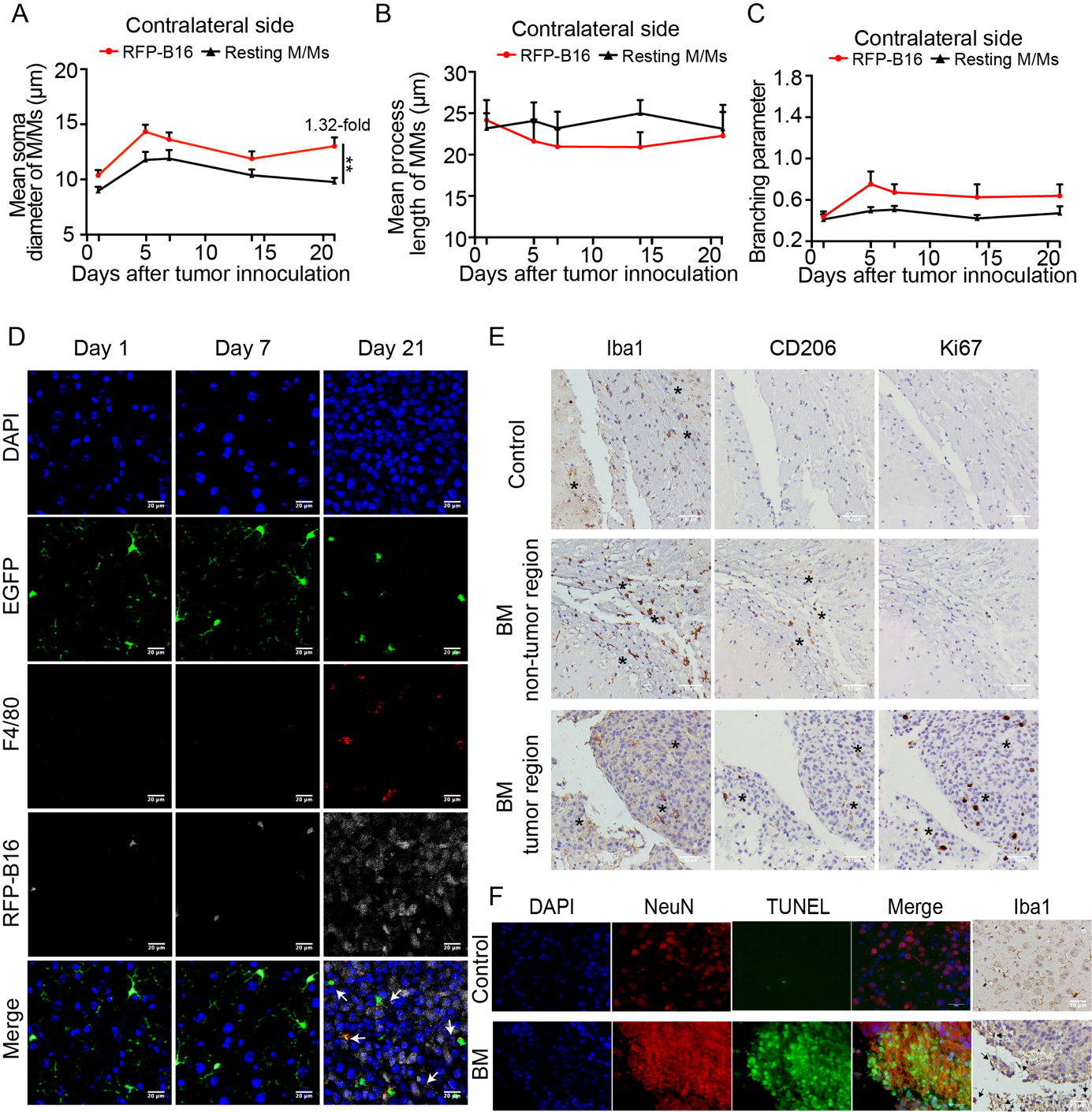


Supplemental Figure 3


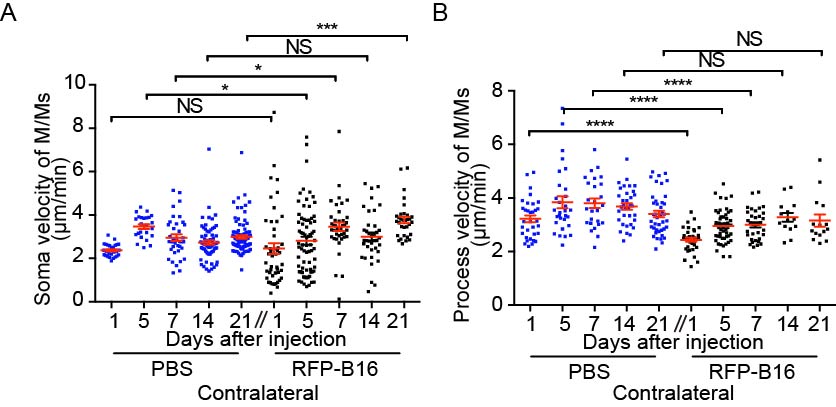


Supplemental Figure 4


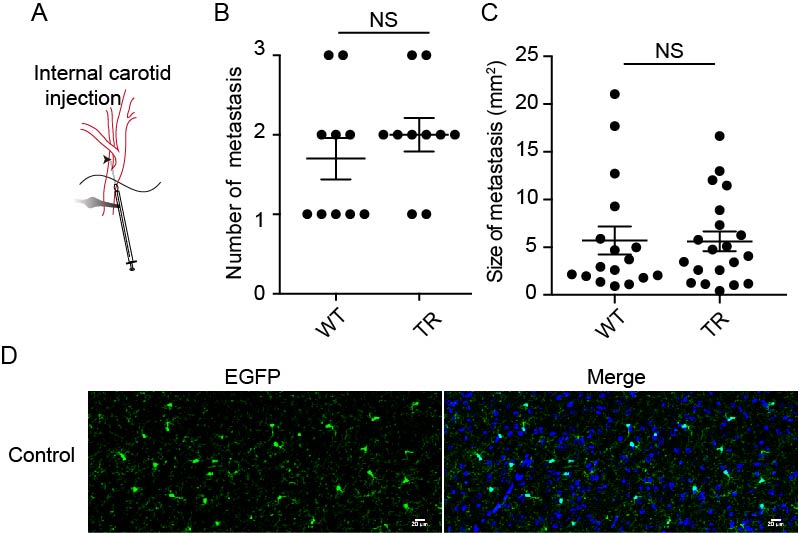


Supplemental Figure 5


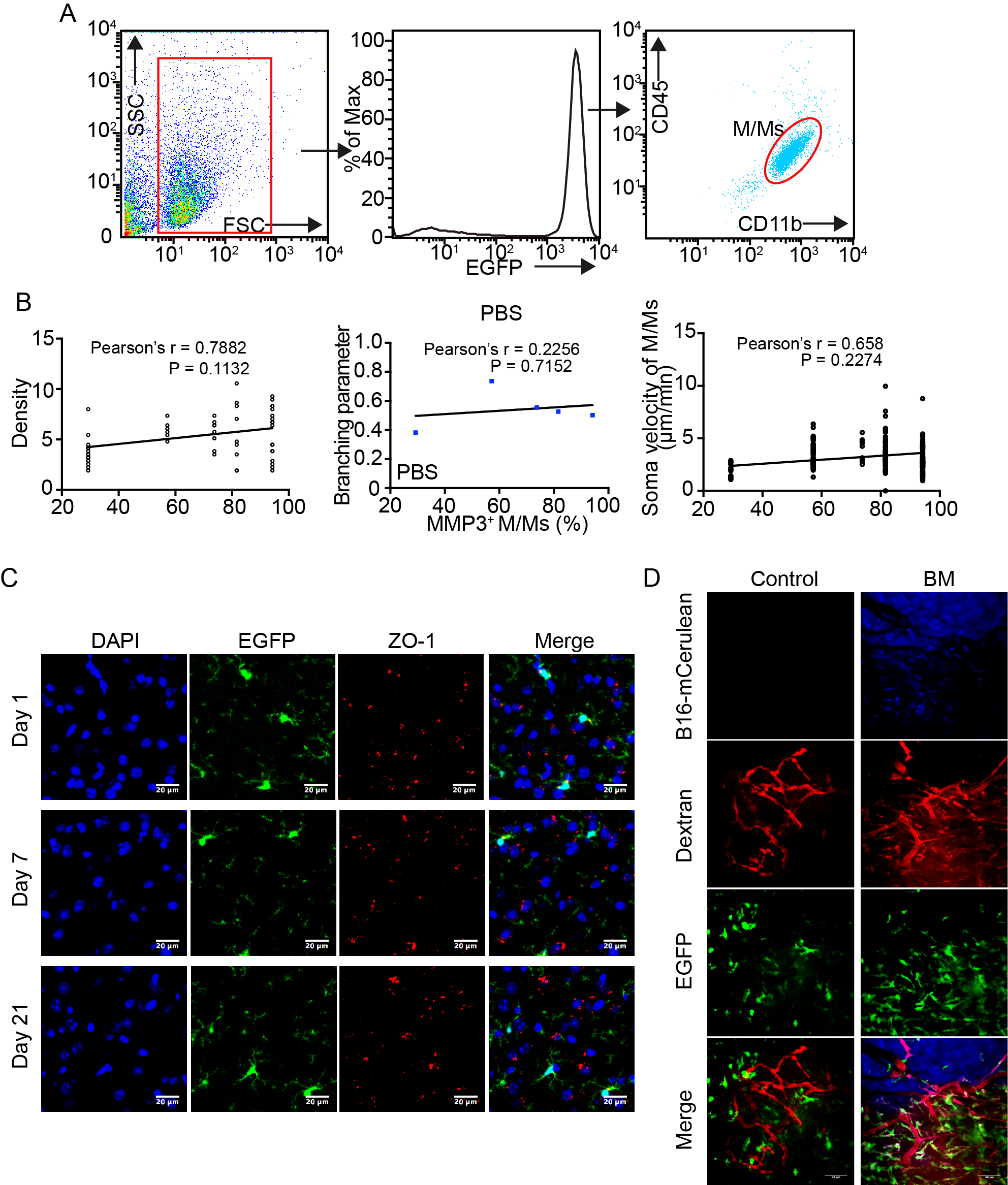

Supplement: Supplementary file 1 — Figure S1. The melanoma brain metastasis established by stereotactically injection. Figure S2. The activation state of M/Ms in melanoma brain metastasis. Figure S3. Velocity of M/Ms after RFP-B16 or PBS injection in the contralateral side. Figure S4. The melanoma brain metastasis established by internal carotid injection. Figure S5. The effect of MMP3 in M/Ms on their activation and the disruption of BBB integrity. (DOCX 14768 kb) [file 12974_2018_1389_MOESM1_ESM.docx]
